# *rprimer*: an R/bioconductor package for design of degenerate oligos for sequence variable viruses

**DOI:** 10.1186/s12859-022-04781-0

**Published:** 2022-06-18

**Authors:** Sofia Persson, Christina Larsson, Magnus Simonsson, Patrik Ellström

**Affiliations:** 1 European Union Reference Laboratory for Foodborne Viruses, Swedish Food Agency, Dag Hammarskjölds väg 56 A, 752 37 Uppsala, Sweden; 2 Department of Medical Sciences, Zoonosis Science Centre, Uppsala University, Uppsala, Sweden; 3 Section of Clinical Virology, Department of Medical Sciences, Uppsala University, Uppsala, Sweden

**Keywords:** Primer design, Degenerate primers, PCR, RT-PCR, qPCR, Digital PCR, Sequence variable virus, Norovirus, Genotyping

## Abstract

**Background:**

This paper presents a new R/Bioconductor package, rprimer, for design of degenerate oligos and PCR assays for sequence variable viruses. A multiple DNA sequence alignment is used as input data, while the outputs consist of comprehensive tables (data frames) and dashboard-like plots. The workflow can be run directly from the R console or through a graphical user interface (Shiny application). Here, rprimer is demonstrated and evaluated by using it to design two norovirus genogroup I (GI) assays: one RT-qPCR assay for quantitative detection and one RT‑PCR assay for Sanger sequencing and polymerase-capsid based genotyping.

**Results:**

The assays generated were evaluated using stool samples testing positive for norovirus GI. The RT-qPCR assay accurately amplified and quantified all samples and showed comparable performance to a widely-used standardised assay, while the RT-PCR assay resulted in successful sequencing and genotyping of all samples. Merits and limitations of the package were identified through comparison with three similar freely available software packages. Several features were comparable across the different tools, but important advantages of rprimer were its speed, flexibility in oligo design and capacity for visualisation.

**Conclusions:**

An R/Bioconductor package, rprimer, was developed and shown to be successful in designing primers and probes for quantitative detection and genotyping of a sequence-variable virus. The package provides an efficient, flexible and visual approach to degenerate oligo design, and can therefore assist in virus research and method development.

**Supplementary Information:**

The online version contains supplementary material available at 10.1186/s12859-022-04781-0.

## Background

Reverse transcription (RT) PCR-based methods are the gold standard for detecting and quantifying viral genetic material in many fields, including clinical diagnostics and food safety control. Successful RT-PCR analysis depends on several factors, but oligo (primer and probe) design is arguably the most critical part of assay development [1]. Poorly designed primers can reduce the efficiency of RT or PCR and poorly designed probes may not hybridise properly with the target, leading to false-negative results or underestimation of the target concentration.

Many viruses, particularly RNA viruses such as norovirus, hepatitis C virus and hepatitis E virus, evolve rapidly and exhibit considerable sequence variability between different strains. This often remarkable sequence diversity results from high mutation rates combined with short generation times and large population sizes. The high mutation rate is mainly explained by the RNA-dependent RNA polymerase (RdRp), which is responsible for the replication of the genome. Unlike many DNA polymerases, RdRps generally have no proofreading activity and are therefore unable to correct errors during replication (a notable exception, however, is the RdRp of coronaviruses), resulting in an average of 10^–6^ to 10^–4^ substitutions per site during replication [2, 3]. However, substitutions are not evenly distributed across the viral genome, as functional interactions between viral proteins and between viral and host proteins limit sequence variation in certain regions. An important challenge in oligo design for viral targets is therefore to identify such conserved regions of the genome, but even the most conserved regions may still have some degree of sequence variability. Furthermore, in various RT-PCR applications (such as genotyping), there is often an interest in amplifying a specific, more variable part of the genome.

A common approach for amplification of sequence variable targets is to use degenerate oligos. A degenerate oligo has at least one position with several possible bases, and the degeneracy refers to the number of unique sequence variants encompassed by the oligo. The degenerate bases are specified by nomenclature determined by IUPAC [4]. For instance, the IUPAC consensus character K can be either G or T, while N can be A, C, G or T. However, as the degeneracy increases, there is a decrease in the fraction of the sequence variant that perfectly matches a specific target sequence and the synthesised PCR products, which reduces the amplification efficiency [5]. Moreover, a larger number of oligos increases the probability of nonspecific interactions with other targets. Thus, keeping the degeneracy as low as possible is often advised, but manually identifying such oligo-binding regions across a large set of target sequences is time-consuming and error-prone. As a result, several strategies and tools have been developed to aid in degenerate oligo design. Examples include GeneFisher [6], Hyden [7], CODEHOP [8], Greene SCPrimer [9], Gemi [10], easyPAC [11] and PrimerDesign [12].

The R language [13] is widely used in programming for bioinformatics. Much of its popularity is due to its versatile package system, which allows users to share software that others can use, modify or implement into their data analysis pipelines. Within the comprehensive R archive network (CRAN) or Bioconductor [14, 15] (the main package repositories for R), two R packages devoted to oligo design (DECIPHER [16, 17] and openPrimeR [18]) are currently available. DECIPHER aims to design primers targeting a specific group of sequences of interest while minimising the potential to cross-react with specified sequences of non-interest, whereas openPrimeR is intended for multiplex primer design. However, neither of these packages supports the design of partially degenerate primers or probe-based assays for real-time PCR or digital PCR (qPCR or dPCR) applications. Partially degenerate primers are an efficient strategy for amplifying highly diverse targets [5]. Moreover, most qPCR and dPCR applications in virology today include fluorescence-labelled hydrolysis probes, because this reduces the risk of false-positive signals compared with fluorescent intercalating dyes.

We developed a new R/Bioconductor package, rprimer, intended to aid in the design of assays with partially or fully degenerate primers, with or without probes. The package identifies oligo binding sites with low to medium degeneracy from a multiple DNA sequence alignment of target sequences of interest. All sequence variants of each oligo are checked for user-specified constraints on length, guanine-cytosine (GC) content, melting temperature (Tm), maximum allowed degeneracy, maximum gap frequency in the target alignment, sequence complexity etc. Our aim in developing the rprimer package was to provide an easy-to-use and efficient approach to generate oligo candidates for variable targets, where sequence conservation analysis forms the basis of the design procedure.

In this study, we evaluated the performance of rprimer in a case study where the package was used to generate two assays targeting norovirus genogroup I (GI), a highly variable RNA virus and common cause of acute gastroenteritis in humans. We developed a RT-qPCR assay for quantitative detection and a RT-PCR assay for Sanger sequencing and polymerase-capsid-based genotyping, and evaluated both assays on clinical samples in the laboratory. Furthermore, we assessed the performance of the rprimer package by comparing it against that of similar, freely available software.

## Implementation

### Overview

**Fig. 1:**
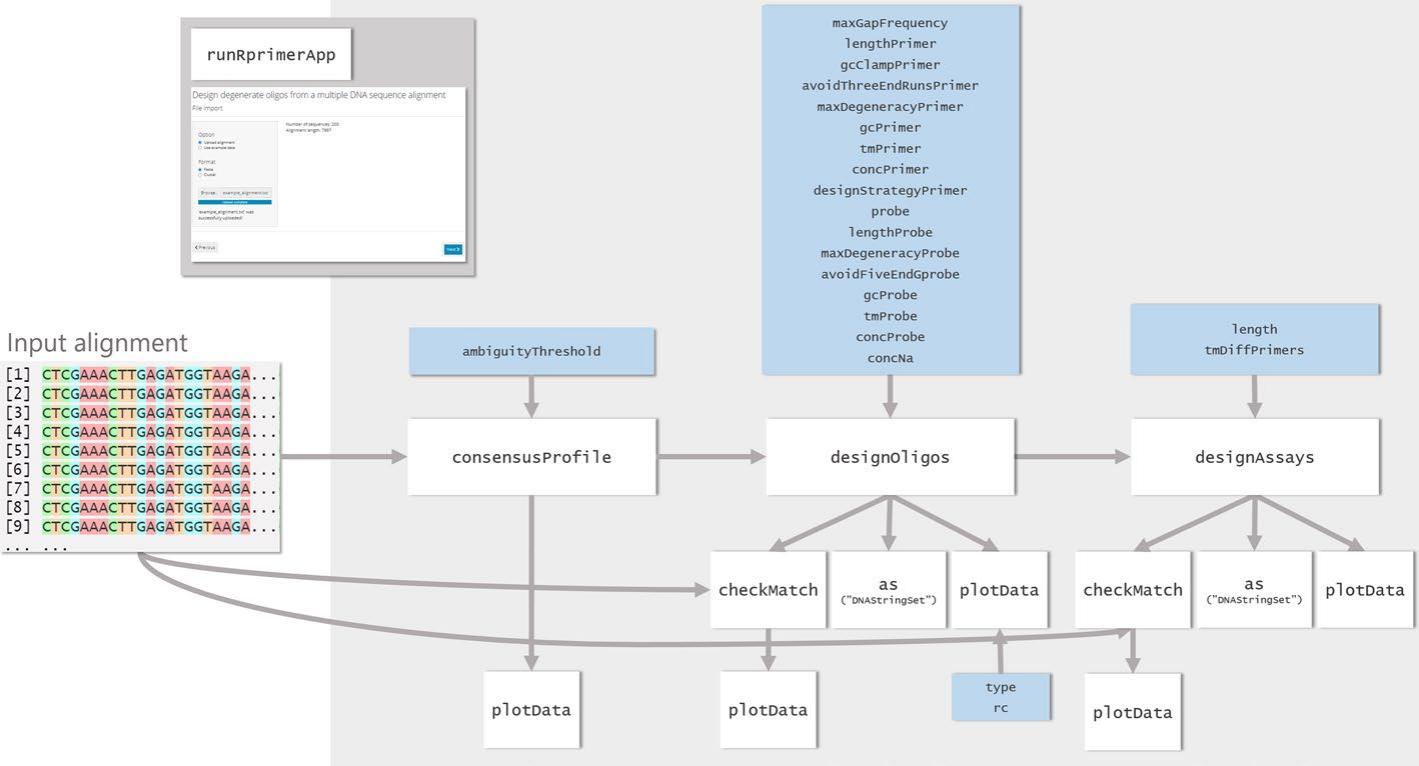
Overview of the rprimer package. White boxes represent functions provided by the package, blue boxes represent settings that the user can modify (if desired; all variables have defaults) and the dark grey box displays the data import page within the Shiny application

**Fig. 2:**
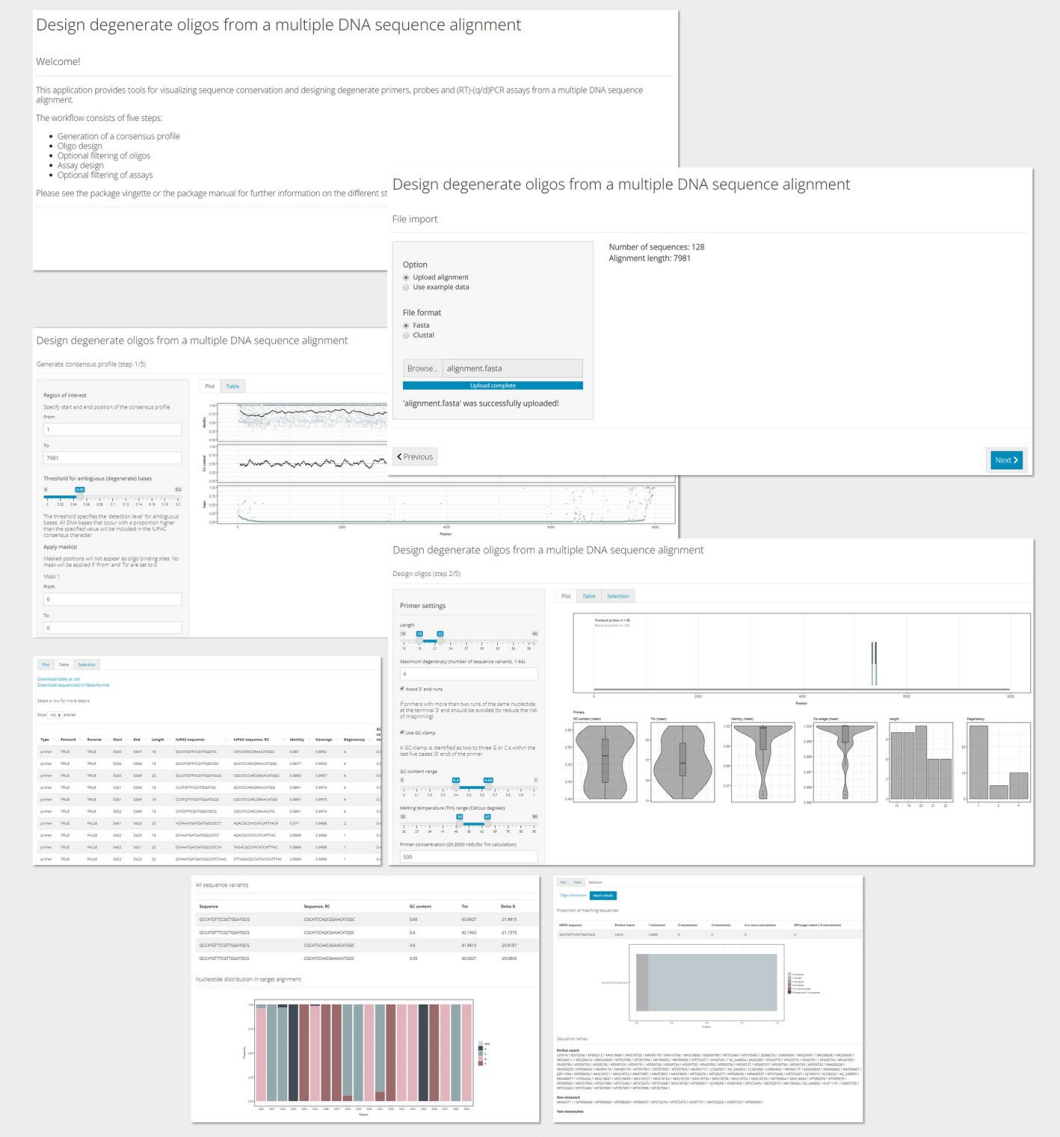
Screenshots from the Shiny application provided by the package. The application is constructed as a wizard that guides the user through the design process and allows them to filter and select oligos and assays to inspect further (not all pages are shown)

The rprimer package generates primers, probes and assays from a multiple DNA sequence alignment with intended target sequences. It can be run directly from the R console or through a graphical user interface (Shiny application)*. *The design workflow consists of three steps: (1) generation of a consensus profile (function name: *consensusProfile*); (2) generation of oligos (*designOligos*); and (3) pairing of oligos to form assays (*designAssays*) (Fig. 1). As a complementary step, it is also possible to investigate how oligos and assays match the input alignment within and outside the intended target binding region (*checkMatch*). All outputs are presented as tabular data and are based on the widely used *DFrame* class [19], and the output from each step can be visualised using a generic plot function (*plotData*). The Shiny application is initialised using *runRprimerApp.* The app interface is constructed as a wizard that guides the user stepwise through the design process and allows them to filter, select and inspect oligos and assays in great detail (see screenshots in Fig. 2).

### Alignment of target sequences and file import

It is up to the user to identify, collect and align target sequences of interest. It is important that the alignment is of good quality and accurately represents the genetic variation in the target population. File import is based on the Biostrings package [20], which is the most widely used R package for importing, storing and manipulating biological sequences. Thus, when using rprimer from the R console, the alignment must be imported using the *readDNAMultipleAlignment* function or converted into a *DNAMultipleAlignment* object before the design process can begin. If desired, it is possible to mask columns (positions) and rows (sequences) using *colMask* and *rowMask* [20]*.* Masked positions will not appear as oligo-binding regions and masked sequences will not be included in the downstream design process. The rprimer package can take from one to several thousand sequences as input.

### Consensus profile

The package workflow starts by generating a consensus profile from the target alignment. This dataset contains all the information needed for the subsequent design process (i.e. gap frequency, majority and IUPAC consensus character etc. at each position). At this point, the user can provide a threshold (ranging from 0 to 0.2) for ambiguous bases and all bases with higher relative frequency than the threshold will be included in the IUPAC consensus character. A value of 0 (default) will capture all variation at each site, but with the potential downside of generating oligos with high degeneracy. A higher value will capture most of the variation, but with the risk of not covering minor variants.

### Oligos

The next step is to design oligos. Primers must be designed, but probes are optional. Primers can be generated using one of two strategies: ambiguous or mixed. The ambiguous strategy uses the IUPAC consensus sequence alone, while the mixed strategy uses both the IUPAC and majority consensus sequence. Thus, the mixed strategy resembles the widely-adopted consensus-degenerate hybrid oligonucleotide primer (CODEHOP) principle [5], and designs primers with a degenerate part at the 3′ end (~ 1/3 of the primer) and a majority consensus part at the 5′ end (~ 2/3 of the primer). The intention is for the degenerate 3′ end to bind specifically to the target sequence in the initial PCR cycles and promote amplification despite potential mismatches at the 5′ consensus end [5]. The PCR products will match the 5′ ends of all primers perfectly, allowing them to be efficiently amplified in later cycles. Probes are always designed using the ambiguous strategy. This is because any mismatch in the probe region may affect the binding of the probe to the template. This may interfere with the digestion of the probe by the 5′-3′ exonuclease activity of the *Taq* DNA polymerase, leading to false-negative results or underestimation of the target concentration.

The oligo design process begins by assembling all possible oligo candidates of user-specified length. The degeneracy is then calculated for each oligo. Oligos with more sequence variants than the user-specified value are removed (the user-specified value can range from 1–64 sequence variants for both primers and probes). Oligos whose binding region has a gap frequency above a user-specified threshold are also removed.

Next, the algorithm generates all possible sequence variants of each oligo. Here, several parameters are calculated for each sequence variant. The user can specify ranges on GC content and melting temperature. For primers, the user can select whether a GC clamp should be used (here identified as the presence of two to three G or C within the five terminal 3′ end bases) and whether primers with more than two mononucleotide repeats (e.g. “AAA”) at the terminal 3′ end should be excluded. For probes, the user can select whether a 5′ end G should be avoided (to prevent quenching of the 5′ fluorophore of hydrolysis probes). All sequence variants of each oligo must fulfil all specified design constraints to be considered as a final candidate. Oligos with at least one sequence variant containing more than four mononucleotide repeats (e.g. “AAAAA”) or more than three dinucleotide repeats (e.g. “ACACACAC”) in a row are not considered.

Melting temperatures are calculated using the nearest-neighbour method [21, 22]. For this, it is possible to specify primer and probe concentration and monovalent cation (Na^+^ and K^+^) concentration in the PCR solution.

### Assays

This step combines oligos to form assays within a desired amplicon length range, specified by the user. If probes are present in the input dataset, only assays with a probe present between the primer pair will be kept. Moreover, if desired, the user can specify a maximum allowed difference between the mean Tm of the forward and reverse primer.

### Scoring and filtering of oligos and assays

All valid oligos are assigned a score, based on the value of the following variables:

- Average identity (degree of conservation at each site in the target alignment, higher is better)
- Average coverage (how well an oligo base covers the variation within the target alignment, higher is better)
- Average GC content (the nearer 0.5, the better)

The best possible score is 0 (i.e. no “penalties”), and the worst possible score is 9. For assays, the average oligo score is shown. Using standard subset methods, it is possible to filter the oligo and assay datasets based upon score and/or other variables of interest.

Importantly, the identity and coverage values differ between ambiguous and mixed primers. For ambiguous primers, they are both calculated from the primer as a whole. For mixed primers, identity is calculated only for the 5′ consensus part and coverage is calculated only for the 3′ degenerate part. This allows for filtering of mixed primer candidates based on both 3′ coverage and 5′ conservation.

### Match check

The *checkMatch* function can be used as a complementary step to the design workflow. It returns the proportion and names of target sequences that match perfectly and those with one, two, three or at least four mismatches to an oligo within the intended oligo binding region in the input alignment (i.e. on-target match). It also gives the proportion and names of target sequences that match with a maximum of two mismatches to at least one sequence variant of the oligo outside the intended oligo binding region (off-target match). The function can be used on both oligo and assay datasets.

### File export and recommended additional analyses

The outputs from *consensusProfile, designOligos, designAssays* and *checkMatch* can be readily exported to.txt or.csv format by standard R functionality (using e.g. *write.table*). To facilitate interoperability with other software, it is also possible to convert the oligos within an oligo or assay dataset to *DNAStringSet* (a widely used format for storing DNA sequences) [20], by using the *as* function (i.e. *as(object, “DNAStringSet”)*). In turn, *DNAStringSet*-objects can be readily exported as fasta-files. When using the Shiny application, the output data can be downloaded as both.csv or fasta-format. Before proceeding to wet laboratory (wet-lab) evaluation, evaluation of the final assays for the potential to form primer-dimers and hairpin structures is highly recommended, as this may severely affect the efficiency of the RT and PCR. These tasks are best performed using well-established software, e.g. OligoAnalyzer (Integrated DNA Technologies).

## Case study: materials and methods

### Target sequence collection and multiple DNA sequence alignment

Norovirus GI genomes were collected by searching for "Norovirus GI"[porgn:__txid122928] on the NCBI nucleotide collection database and filtering for sequence lengths > 7000 bases (the complete norovirus GI genome is approximately 7500 bases). All sequences identified (n = 134) were genotyped using Norovirus Typing Tool Version 2.0 [23] to verify that they were correctly classified as norovirus GI. Six sequences were removed because they contained long stretches of “N”. The remaining 128 sequences were aligned using the online resource of mafft version 7 [24], with Auto settings.

### Additional checks on final assays

Before wet-lab evaluation, the final assay candidates were checked in silico for primer-dimer formation, hairpin structures and non-norovirus GI (i.e. off-target) interaction. The potential to form primer-dimer and hairpin structures was assessed using OligoAnalyzer Version 3.1 (Integrated DNA Technologies). Non-norovirus GI interactions were identified by using BLAST [25] to search for matches to everything except norovirus GI in the NCBI nucleotide collection database.

### Viruses

Ten human stool sample suspensions previously identified as testing positive for norovirus GI by RT-qPCR (at Uppsala University Hospital) were used for assay evaluation. Ribonucleic acid was extracted with a NucliSENS miniMAG instrument and NucliSENS magnetic extraction reagents (BioMérieux), with 60 µl sample volume and 120 µl elution volume.

### Primers and probes

Primers were purchased from Integrated DNA Technologies and probes were purchased from Life Technologies. All primers and probes are listed in Table 1. Note that two assays were used in RT-qPCR: an assay developed in this study and a reference assay previously described [26].

**Table 1:** Primers and probes used in this study

Assay name	Application	Type	Sequence (5′-3′)	Sense	Intended binding region in the norovirus GI reference sequence (NC_001959.2)	References
A	Detection	Forward primer	GCCATGTTCCGCTGGATG	Plus	5282–5299	This study
A	Detection	Reverse primer	CGTCCTTAGACGCCATCATCATTTAC	Minus	5354–5379	This study
A	Detection	Probe	[FAM]-CGRTCTCCTGTCCACA-[MGB-EQ]	Minus	5319–5334	This study
Reference	Detection	Forward primer	CGCTGGATGCGNTTCCAT	Plus	5291–5308	[34, 26]
Reference	Detection	Reverse primer	CCTTAGACGCCATCATCATTTAC	Minus	5354–5376	[33, 26]
Reference	Detection	Probe	[FAM]-TGGACAGGAGATCGC-[MGB-EQ]	Plus	5321–5335	[35, 26]
B	Typing, amplification	Forward primer	CTTCACAGGTGAACAGCATAAAYCAYTGG	Plus	4758–4786	This study
B	Typing, RT and amplification	Reverse primer	CATGTTGCCAACCCAACCRTTRTACA	Minus	5653–5678	This study
B	Typing, sequencing	Forward primer	CTTCACAGGTGAACAGC	Plus	4758–4774	This study
B	Typing, Sequencing	Reverse primer	CATGTTGCCAACCCAACC	Minus	5661–5678	This study

FAM, 6-carboxyfluorescein; MGB-EQ, minor groove binder-eclipse quencher

### RT-qPCR

One-step RT-qPCR was performed with the TaqPath 1-Step RT-qPCR Master Mix CG (Thermo Fisher Scientific) on a LightCycler 96 System (Roche, Basel, Switzerland). Each reaction contained 500 nM forward primer and 900 nM reverse primer. The probe concentration was 150 nM for the assay developed in this study and 250 nM for the reference assay. For each sample, a total of 25 μl reaction mix was prepared with 20 μl of reagents and 5.0 μl of sample. RT-qPCR was performed with RT at 50 °C for 15 min, inactivation of the reverse transcriptase and activation of the DNA polymerase at 95 °C for 2 min, followed by 45 cycles of denaturation at 95 °C for 3 s, annealing and elongation at 60 °C for 30 s. Cycle of quantification (Cq) values were determined by the LightCycler 96 software, version 1.1 (Roche). Quantification was performed using a tenfold dilution series of linearised plasmid DNA with a norovirus GI insert, as previously described [27]. The standard curve ranged from 5·10^5^ to 50 plasmid equivalents per reaction. All samples and controls were run in duplicate wells. Stool sample eluates were diluted 10 times before RT-qPCR. Results were analysed with the LightCycler® 96 software, version 1.1 (Roche) and amplification curve plots were generated using the ggplot2 [28] package in R. A 95% confidence interval (CI) for the average difference in (log) quantity between the assay developed in this study and the reference assay was obtained from a two-tailed, paired *t*-test. Data from the RT-qPCR experiment is provided in Additional file 1 and a complete minimum information for quantitative real-time PCR experiments (MIQE) checklist is provided in Additional file 2.  

### RT-PCR and Sanger sequencing

One-step RT-PCR was performed with SuperScript IV One-Step RT-PCR System (Invitrogen) on a T100 Thermal Cycler (Bio-Rad). Each reaction contained 500 nM forward primer and 500 nM reverse primer. For each sample, a total of 25 µl reaction mix was prepared, with 20 µl of reagents and 5.0 µl of sample. RT-PCR was performed with RT at 50 °C for 10 min, inactivation of the reverse transcriptase and DNA polymerase activation at 98 °C for 2 min, followed by 10 cycles of denaturation at 98 °C for 10 s, annealing at 50 °C for 10 s, elongation at 72 °C for 30 s and 30 cycles of denaturation at 98 °C for 10 s, annealing at 60 °C for 10 s and elongation at 72 °C for 30 s. A final extension step was performed at 72 °C for 5 min. The RT-PCR products were visualised on a FlashGel System (Lonza), and purified using ExoSAP-IT PCR Product Cleanup Reagent (Thermo Fischer Scientific). Sanger sequencing was performed at Eurofins Genomics in Germany, using the sequencing primers in Table 1. All sequences obtained were genotyped using Norovirus Typing Tool version 2.0 [23].

### Package performance assessment

The time taken to perform each step of the design process was measured using the microbenchmark package [29] in R. The analysis was performed on a Lenovo ThinkPad laptop with an Intel Core i5-8265 processor with 16.0 GB RAM, with a Windows 10 operating system. Each step was repeated 20 times. Plots were generated using the ggplot2 package [28] in R.

### Comparison with other software

To identify strengths and weaknesses with rprimer, three other freely available tools (Gemi [10], DECIPHER [16, 17], and openPrimeR [18]) were selected for comparison. Gemi was identified from a review of primer design programs published in 2020 [30]. It was selected because it is intended for similar purposes and uses the same overall strategy as rprimer (i.e. based on a multiple DNA alignment, but designs degenerate oligos from a consensus sequence from the alignment). DECIPHER and openPrimeR were selected because they are R packages, but with somewhat different purposes and strategies than rprimer: DECIPHER aims to identify primers targeting a specific group of sequences of interest while minimising the potential to cross-react with specified sequences of non-interest, whereas openPrimeR is intended to multiplex primer design.

Gemi was downloaded from https://sites.google.com/site/haithamsobhy/software. DECIPHER was run online at http://www2.decipher.codes/DesignPrimers.html and locally through the R console (by downloading the package from Bioconductor). openPrimeR was run locally as a docker image by following the instructions at https://github.com/matdoering/openPrimeR. All web pages were accessed on January 10, 2022. For Gemi and DECIPHER, alignment with 128 norovirus GI sequences was used as input, whereas the corresponding unaligned sequences were used for openPrimeR. openPrimeR offers two different strategies for primer design: 'naive' and 'tree'. The naive algorithm constructs primers from substrings of the input target sequences, while the tree algorithm initialises primers through alignment of the input target sequences followed by hierarchical clustering and tree construction. We selected the tree algorithm because it designs degenerate primers and was recommended in the reference manual for our type of application (related sequences).

## Results and discussion

### Case study

To illustrate and evaluate the functionality of rprimer, two assays targeted at norovirus GI were designed.

### Assay A: RT-qPCR for quantitative detection of norovirus GI

**Fig. 3:**
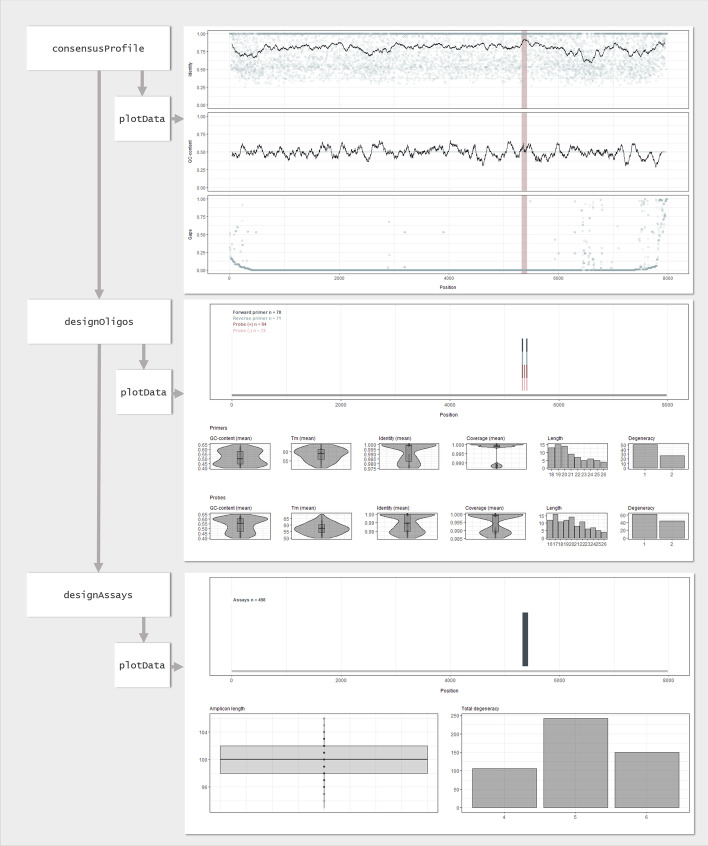
Visual summary of the design of Assay A. The workflow and function calls are displayed to the left and the output from the generic *plotData* function to the right. Top: Consensus profile from the input alignment. High identity in combination with low gap proportion indicate high sequence conservation. Dots show the value at each position and black lines represent centred running averages. The highlighted area indicates target regions of the candidate assays (added after the design was completed). Middle: All oligo candidates. Bottom: All assay candidates

**Fig. 4:**
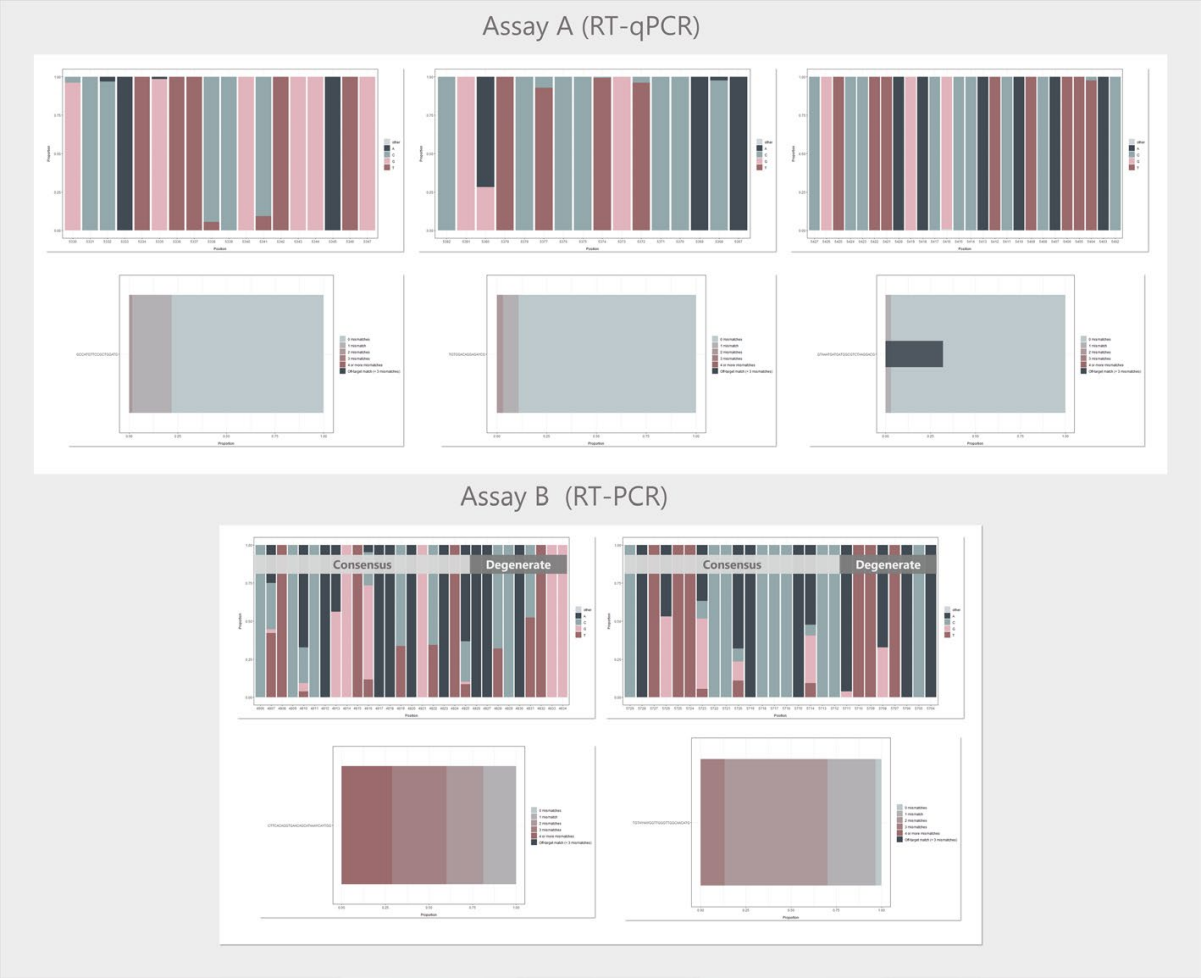
Binding region and match information for Assay A and Assay B. For each assay, the upper plots indicate the nucleotide distribution in the target alignment within the oligo binding regions (left: forward primer; middle: probe; right: reverse primer). The sequence is shown in the same direction as the oligo (i.e. in oligo 5′-3′ direction). The lower plots show the proportion of target sequences in the input alignment that matches perfectly, or with one, two, three or four or more mismatches to the oligo within the intended binding region (on-target match). They also show the proportion of target sequences that match (with maximum of two mismatches) to at least one sequence variant of the oligo outside the intended binding region (off-target match). Assay B was designed using the mixed strategy and the consensus and degenerate part of the primer is indicated. The plots were generated using the *plotData* function

The first task was to design a broadly reactive RT-qPCR assay for quantitative detection. For this, we decided to use the ‘ambiguous’ primer design strategy with strict settings for degeneracy (no more than two variants for both primers and probes), but with a high threshold for ambiguous bases (10%). We had to shorten the minimum probe length to 16 to find any potential assays (the default length is 18–22). These options resulted in 498 assay candidates, all within a conserved stretch from position 5324 to 5429 in the input alignment (corresponding to position 5276 to 5381 in the norovirus GI reference sequence, NC_001959.2) (Fig. 3). To select a final candidate, we first filtered the design output dataset upon score, which reduced the number of assays to 24. For these, we ran *checkMatch* to identify the best candidates regarding both on- and off-target matches. Figure 4 shows the nucleotide distribution at the oligo binding regions of the selected final assay (Assay A), together with the proportion of matching and mismatching target sequences.

**Fig. 5:**
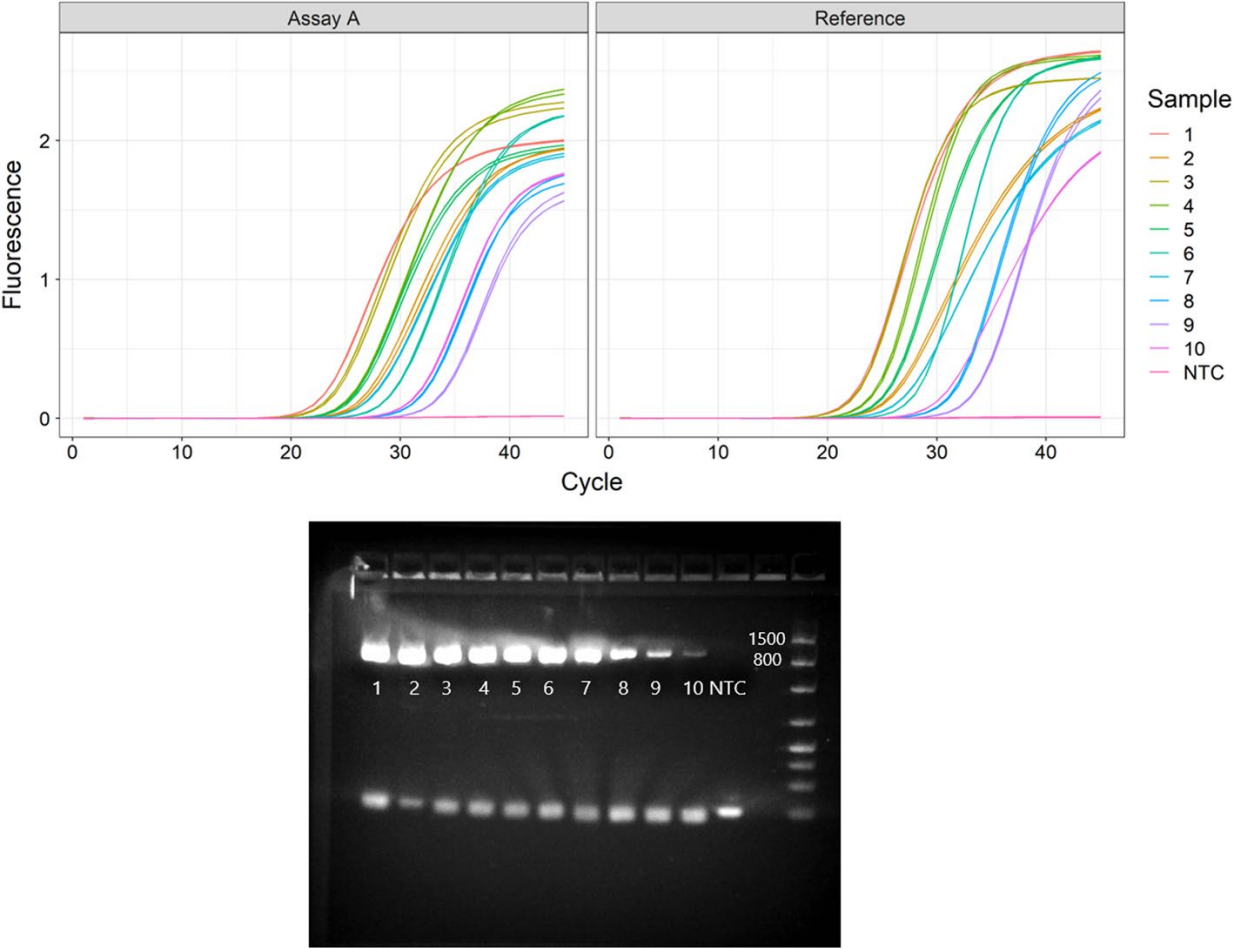
Evaluation of assay performance using 10 stool samples testing positive for norovirus GI. Top: Fluorescence data from RT-qPCR with Assay A (designed using rprimer), in comparison to a reference assay. Each sample was run in duplicate wells. Bottom: Gel picture following RT-PCR with Assay B (designed using rprimer). NTC: no template control. The intended amplicon size is 924 bp

We evaluated Assay A together with a standardised reference assay (Table 1) [26] on 10 stool samples previously identified as testing positive for norovirus GI. All samples were successfully amplified and quantified using both assays. The assays agreed well in quantification: the estimated concentration was on average 1.1 times higher (95% CI 0.89–1.3 times) with Assay A than with the reference assay (Table 2). Both assays provided acceptable sigmoidal-shaped amplification curves (Fig. 5). Based on the standard curve, the amplification efficiency was 105% for Assay A and 92% for the reference assay (for a well-performing qPCR assay, the value often lies between 95 and 105% [1]). The *R*^*2*^ value was 1.00 for both assays (0.98 or higher is considered acceptable [1]), and the *y*-intercept (the Cq value corresponding to 1 copy/µl in the sample well) was 40.20 for Assay A and 40.72 for the reference assay.

**Table 2:** Quantification and genotyping results from the case study

Sample number	Assay A: RT-qPCR based quantification			Assay B: RT-PCR based genotyping	
	Quantity (copies/ml), assay A	Quantity (copies/ml), reference assay (ISO 151216-1)	Bias (quantity from assay A/quantity from reference assay)	Capsid type	Polymerase type
1	2.00E+09	1.08E+09	1.85	GI.P13 (GI.Pd)	GI.3
2	1.19E+08	1.27E+08	0.94	GI.P1	GI.1
3	1.07E+09	9.83E+08	1.09	GI.P7	GI.7
4	2.85E+08	3.43E+08	0.83	GI.P4	GI.4
5	2.44E+08	1.71E+08	1.43	GI.P13 (GI.Pd)	GI.3
6	2.33E+07	2.62E+07	0.89	GI.P4	GI.4
7	7.95E+07	5.56E+07	1.43	GI.P4	GI.4
8	3.25E+06	3.27E+06	0.99	GI.P13 (GI.Pd)	GI.3
9	8.89E+05	9.51E+05	0.93	GI.P13 (GI.Pd)	GI.3
10	4.72E+06	5.77E+06	0.82	G1.P2	GI.2

The conserved region described above constitutes the target for most previously published RT-qPCR assays for norovirus GI (e.g. [31–35]. To the best of our knowledge, no previously published assay is identical to Assay A, although some oligos/assays differ by only a few bases.

### Assay B: RT-PCR and Sanger sequencing for genotyping of norovirus GI

The next task was to design a conventional RT-PCR assay for genotyping. Norovirus genotyping is ideally performed by sequencing parts of both the polymerase and capsid gene. From an analysis of publicly available sequences, we identified a minimum region of interest (ROI) required to obtain reliable genotyping results. We masked all positions except for 1000 nucleotides on each side of the ROI in the target alignment. The remaining potential primer binding sites were highly variable, and hence we opted for the ‘mixed’ primer design strategy. To provide sufficiently high melting temperature despite any potential 5′ end mismatches, we decided to design relatively long primers (25–40 nt). The maximum allowed degeneracy was set to four, and we opted for amplicon lengths of 300–1000 base pairs.

In the assays generated, we filtered for primers with high 3′ coverage (provided by the coverage variable in the output dataset) and high 5′ conservation (provided by the identity variable). The binding regions of the final candidate primers (Assay B) are shown in Fig. 4. The average coverage was 1 for both the forward and reverse primer, meaning that their 3′ ends matched perfectly to all sequences in the input alignment. Consequently, all mismatches shown in Fig. 4 are located within the 5′ end consensus part of the primers.

To enable amplification of mismatching sequences in the initial amplification rounds, we used a PCR protocol with an annealing temperature of 50 °C for the first 10 cycles, followed by 60 °C for the remaining 30 cycles. We used the consensus parts of the PCR primers as sequencing primers (Table 2) since they matched the amplified products perfectly. All samples were successfully amplified, sequenced and genotyped with Assay B (Fig. 5 and Table 2).

We identified two potential limitations with Assay B. First, one of the sequence variants of the reverse primer is self-complementary at the six terminal bases at the 3′ end (the change in Gibb’s free energy, delta G, for the formation is − 7.12 kcal/mol, as estimated by OligoAnalyzer 3.1). This probably causes primer-dimers (see the gel picture in Fig. 5), which can lower the RT and PCR efficiency. Second, 29% of the target sequences in the input alignment mismatched, with at least four mismatches to the consensus part of the forward primer (Fig. 4). Further optimisation, such as nesting, may therefore be needed for use on low-concentration targets. Overall, this underlines a difficulty in global primer design towards sequence variable targets, i.e. it is not always possible to find conserved binding regions that fulfil all desired design criteria.

We could not find any previous publication using primers identical to those in Assay B. However, one assay for polymerase-capsid-based genotyping of norovirus GI, described by van Beek et al*.* [36], covers position 4499–5683 of the norovirus GI reference sequence (NC_001959.2). Assay B covers position 4758–5678.

### Package performance

**Fig. 6:**
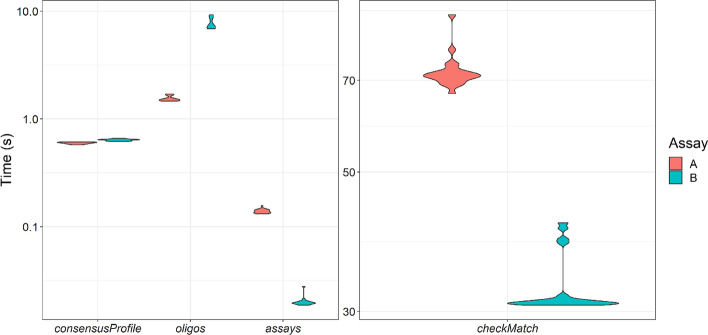
Evaluation of rprimer package performance. The violin plots show the time required to perform each step of the oligo design process in assay A and B, on a generic workplace laptop using a multiple DNA sequence alignment of 128 norovirus GI sequences. The *checkMatch* function was run with all oligo candidates (A, n = 184, B, n = 128) towards 128 target sequences. Each step was repeated 20 times

To assess package performance, we estimated the time required for a generic workplace laptop to design Assay A and B. Both tasks were completed within a few seconds (Fig. 6, left). We also estimated how long it took to perform *checkMatch* (a complementary step to the workflow) on all oligos generated from each design process (A, n = 184, B, n = 128) towards all 128 target sequences. The median time to run *checkMatch* was 1 min and 8 s for A and 31 s for B (Fig. 6, right).

These tests were performed on oligos with low degeneracy (a maximum of two variants per oligo was allowed for Assay A and four for Assay B). When higher degeneracy is selected, the time to generate oligos will increase because more regions will qualify as binding sites, and the parameters (Tm, GC-content etc.) will be calculated for more sequence variants. To illustrate this, we changed the degeneracy to 64 (the highest possible value) and re-ran *designOligos*. For this task, the median time to generate oligos was 20 s for assay A (instead of 1.5 s when the degeneracy was two; Fig. 6) and 4 min and 10 s for assay B (instead of 7.2 s when the degeneracy was two; Fig. 6). With a maximum degeneracy of 64, 691 oligo candidates were identified for A and 4971 for B.

### Comparison with other software, strengths and limitations

Direct comparisons with other software are difficult. Many available programs are highly specialised to address specific needs, and there was an inevitable risk of bias when comparing a tool we developed ourselves with tools that we had not used previously. With these limitations in mind, we assessed rprimer together with three other freely available programs: Gemi [10], DECIPHER [16, 17], and openPrimeR [18] (the two latter are R packages). We imported all 128 norovirus GI sequences used for designing the assays in this study, in the format requested by the respective software. To obtain comparable results, we tried to mimic the design settings used for Assay A as much as possible. We did not attempt to replicate the design of Assay B because none of the other programs provided the option to generate partially degenerate primers.

Gemi and DECIPHER designed assays rapidly (within a few seconds) and identified the same region as Assay A as the top candidate (although designing assays with probes was not possible with DECIPHER). For DECIPHER, the top-scoring forward primer was identical to Assay A and the reverse primer differed by only a few bases. For our purposes, both Gemi and DECIPHER were less extensive than rprimer, but enabled specification of e.g. primer and amplicon length and the maximum number of degenerate positions (Gemi) or degeneracy (DECIPHER, referred to as permutations).

openPrimeR is also designed to sequence variable targets and aims to find the minimal number of multiplex compatible primers required to amplify all specified target sequences of interest. rprimer is not designed to generate multiplex primers and does not attempt to minimise the number of primers in the same sense. Instead, it calculates the degeneracy of each oligo obtained from the IUPAC consensus sequence, which may result in oligos with redundant sequence variants (i.e. full coverage could have been achieved with fewer sequence variants). In addition, openPrimeR is more extensive than rprimer and evaluates the potential for e.g. self-dimerisation and secondary structure formation.

With openPrimeR, we first attempted to design primers from all 128 (unaligned) full-length genomes, but interrupted the process after several hours. Based on this, we decided to design only forward primers, from a subset of five randomly selected genomes (and only from a 500 nt region covering the most conserved part of the genome). We decided to allow a maximum of mismatches and not more than two primer sequence variants for full coverage. The process took about 10 min and the top scoring primer covered position 5318–5335 in the norovirus reference sequence (NC_001959.2), which is similar to the probe of Assay A (position 5319–5334, Table 1). However, it should be noted that openPrimeR (with the option we selected) aligns target sequences as part of the primer design procedure, whereas our software uses aligned sequences as input.

A strength of rprimer lies in its simplicity - the approach to oligo and assay design is intuitive and widely established. The workflow consists of several steps and the user has complete control over the parameters that constrain the design. When no oligos or assays are generated, the user can simply go back and adjust (relax) the parameters. To verify that the oligos have been generated correctly, the user can check how the oligos match each target sequence (*checkMatch*) and examine the intended binding region in the input alignment (*plotData*; see Fig. 4).

Compared with other software, additional strengths of rprimer are its capacity for visualisation (exemplified in Figs. 3, 4) and its flexibility in oligo design. For instance, rprimer can design assays with probes (possible with Gemi, but not with the two other programs), provides the possibility to set a threshold value for degenerate bases (not possible with any of the three programs) and gives two options for primer design (fully or partially degenerate, not possible with any of the three programs). By using partially degenerate primers, we were able to amplify and sequence a highly variable region of the norovirus GI genome. Moreover, both openPrimeR and DECIPHER recommend/require external third-party software, while rprimer has no such dependencies, which makes installation easier.

The most apparent limitation with rprimer is the inability to check for primer-dimer and hairpin formation, which openPrimeR can do. Moreover, rprimer checks for off-target interactions within the target alignment but cannot analyse them in reference datasets, which DECIPHER can do. To tackle these limitations, we made great efforts to select the most widely-adopted data structures as inputs and outputs, to promote interoperability with other software capable of performing these or other additional analyses.

### Intended use

In addition to norovirus GI, we have used rprimer to develop oligos and assays for several viral species with different sequence variability, ranging from those with moderate variability (e.g. hepatitis A virus) to extremely high variability (e.g. hepatitis C virus). We did not evaluate these assays in the laboratory, but compared them with previously published assays. This showed that the assays generated by rprimer were similar to several widely used assays, confirming the accuracy of rprimer (unpublished data). Note, however, that rprimer is not recommended for highly conserved target species, and we have not evaluated the tool for other targets than viruses.

### Future development

For future versions of rprimer, we plan to implement calculations of delta G for possible self- and heterodimer structures for candidate oligos and assays, as an integral part of the oligo design process. We also intend to further evaluate and update the oligo and assay scoring or filtering system.

## Conclusions

This study showed that the novel R/Bioconductor package rprimer can effectively design primers, probes and RT-(q)PCR assays to detect, quantify and sequence a highly variable RNA virus. The package was successful in identifying the most conserved part of the genome to act as an RT-qPCR assay target and in finding more difficult primer-binding sites to amplify a highly variable region. Comparison with similar freely available software revealed that rprimer provides a flexible and visual approach to degenerate oligo design. It can thus be helpful for diagnostic method development and studies on sequence variable viruses.

## Availability and requirements

- Project name: rprimer.
- Project home page: https://www.bioconductor.org/packages/rprimer.
- Operating system(s): Tested on Windows, MacOS and Ubuntu.
- Programming language: R 4.2 or higher.
- Other requirements: None.
- License: GPL-3.
- Any restrictions to use by non-academics: See licence.

## Supplementary Information

**Additional file 1**. Data from the RT-qPCR experiment. 

**Additional file 2**. MIQE checklist. 

## Data Availability

The source code to rprimer is openly available at https://github.com/sofpn/rprimer. RT-qPCR data, sequences and R code generated in this study are openly available at https://github.com/sofpn/rprimer-paper.

## Abbreviations

CI: Confidence interval
CODEHOP: Consensus-degenerate hybrid oligonucleotide primer
Cq: Cycle of quantification
CRAN: Comprehensive R Archive Network
delta G: Change in Gibb’s free energy
dPCR: Digital polymerase chain reaction
FAM: 6-Carboxyfluorescein
G: Genogroup
GC: Guanidine cysteine
IUPAC: International Union of Pure and Applied Chemistry
MGB-EQ: Minor groove binder-eclipse quencher
NCBI: National Centre for Biotechnology Information
nt: Nucleotides
NTC: No template control
PCR: Polymerase chain reaction
RC: Reverse complement
ROI: Region of interest
RT: Reverse transcription
RT-PCR: Reverse transcription-polymerase chain reaction
RT-qPCR: Reverse transcription-quantitative polymerase chain reaction
Tm: Melting temperature
qPCR: Quantitative polymerase chain reaction
